# Prospective validation of 18F-Fluoroethylcholine as a tracer in PET/MRI for the evaluation of breast lesions and prediction of lymph node status

**DOI:** 10.1007/s11547-023-01633-6

**Published:** 2023-05-23

**Authors:** Paola Clauser, Sazan Rasul, Panagiotis Kapetas, Barbara J. Fueger, Ruxandra-Iulia Milos, Theresa Balber, Neydher Berroterán-Infante, Marcus Hacker, Thomas Hans Helbich, Pascal Andreas Thomas Baltzer

**Affiliations:** 1 Department of Biomedical Imaging and Image-Guided Therapy, Division of General and Pediatric Radiology, Medical University of Vienna, Währinger Gürtel 18-20, 1090 Vienna, Austria; 2 Department of Biomedical Imaging and Image-Guided Therapy, Division of Nuclear Medicine, Medical University of Vienna, Vienna, Austria; 3 Department of Biomedical Imaging and Image-Guided Therapy, Division of Molecular and Structural Preclinical Imaging, Medical University of Vienna, Vienna, Austria

**Keywords:** Breast neoplasms, Fluoroethylcholine, Lymph nodes, Magnetic resonance imaging positron-emission tomography

## Abstract

**Purpose:**

To assess 18F-Fluoroethylcholine (18F-FEC) as a PET/MRI tracer in the evaluation of breast lesions, breast cancer aggressiveness, and prediction of lymph node status.

**Materials and methods:**

This prospective, monocentric study was approved by the ethics committee and patients gave written, informed consent. This clinical trial was registered in the EudraCT database (Number 2017-003089-29). Women who presented with suspicious breast lesions were included. Histopathology was used as reference standard. Simultaneous 18F-FEC PET/MRI of the breast was performed in a prone position with a dedicated breast coil. MRI was performed using a standard protocol before and after contrast agent administration. A simultaneous read by nuclear medicine physicians and radiologists collected the imaging data of MRI-detected lesions, including the maximum standardized 18F-FEC-uptake value of breast lesions (SUV_maxT_) and axillary lymph nodes (SUV_maxLN_). Differences in SUV_max_ were evaluated with the Mann–Whitney U test. To calculate diagnostic performance, the area under the receiver operating characteristics curve (ROC) was used.

**Results:**

There were 101 patients (mean age 52.3 years, standard deviation 12.0) with 117 breast lesions included (30 benign, 7 ductal carcinomas in situ, 80 invasive carcinomas). 18F-FEC was well tolerated by all patients. The ROC to distinguish benign from malignant breast lesions was 0.846. SUV_maxT_ was higher if lesions were malignant (*p* < 0.001), had a higher proliferation rate (*p* = 0.011), and were HER2-positive (*p* = 0.041). SUV_maxLN_ was higher in metastatic lymph nodes, with an ROC of 0.761 for SUV_maxT_ and of 0.793 for SUV_maxLN._

**Conclusion:**

Simultaneous 18F-FEC PET/MRI is safe and has the potential to be used for the evaluation of breast cancer aggressiveness, and prediction of lymph node status.

## Introduction

Breast cancer remains a leading cause of morbidity and mortality in women [1]. Critical research gaps in breast cancer diagnosis and treatment are the lack of validated imaging biomarkers that can provide minimally invasive diagnosis and reliable information about breast cancer aggressiveness and metastatic potential [2]. Therapeutic decisions are based largely on the results of image-guided biopsies. During biopsy, only part of the lesion or lymph node is sampled and relevant information might be missed, i.e., invasive components or lymph node metastasis; further, lesion aggressiveness might be underestimated [3, 4]. This could result in *undertreatment*. Thus, imaging tests that could not only distinguish benign from malignant findings, but also characterize breast cancer aggressiveness, including lymph node status, are desirable.

Abnormal choline metabolism plays a pivotal role in breast cancer cells [5]. Increased choline kinase-α activity has been observed in breast cancer and is associated with malignant progression [5, 6]. The assessment of choline kinase-α activity could provide valuable diagnostic and prognostic information for breast cancer diagnosis and treatment [7].

To detect these metabolic alterations, radiolabeled choline tracers have been developed, which trace cellular choline transport and phosphorylation in vivo [8]; one of the most commonly used choline tracers is 18F-Fluoroethylcholine (18F-FEC) [8]. 18F-FEC has been proven safe in humans and is able to detect primary and secondary malignant lesions in prostate cancer [8, 9]. In vitro studies have confirmed a detectable increase in choline metabolism in other cancer cells as well [10]. The role of 18F-FEC in breast lesions has been addressed by only few studies, which presented several limitations and included only malignant lesions [7, 11, 12]. A single work on 39 breast lesions showed the potential of 18F-FEC to differentiate benign from malignant breast lesions [13]. The full potential of 18F-FEC as an imaging biomarker for diagnostic purposes and prediction of breast cancer aggressiveness remains elusive.

The aim of this prospective, diagnostic, validation study was to assess the role of 18F-FEC as a radiotracer in the evaluation of breast lesions, breast cancer aggressiveness, and prediction of lymph node status in simultaneous PET/MRI.

## Materials and methods

This prospective, single-center EudraCT-registered (2017-003089-29) diagnostic study was approved by the national authorities and the local ethics committee. All patients gave written, informed consent. The study was performed in accordance with the Declaration of Helsinki statement for medical research involving human subjects.

Inclusion criteria were breast lesions classified as suspicious on conventional imaging (ACR BI-RADS 4 or 5 on mammography, tomosynthesis, and/or ultrasound); age above 18 years; and availability of histopathological confirmation of the lesion.

Exclusion criteria were unstable or non-compliant patients; pregnant or breast-feeding patients; radiation therapy or chemotherapy within the last 6 months or surgical interventions less than 12 weeks before the PET/MRI examination; known contraindications to MRI and/or the intravenous administration of gadolinium; renal insufficiency. Data were collected on adverse events that occurred during or after the examination.

### PET-MRI of the breast

The examinations were performed using a simultaneous whole-body combined PET/MRI device (Biograph mMR system, Siemens, Erlangen, Germany), characterized by an MRI-compatible PET detector integrated with a 3.0 Tesla MRI scanner.

Radiosynthesis of 18F-Fluoroethylcholine followed a two-step reaction procedure using a remote-controlled synthesizer (Nuclear Interface, GE Healthcare, Uppsala, Sweden). 18F-Fluoride was produced on-site using [^18^O]H_2_O and a medical cyclotron via an ^18^O(p,n)^18^F reaction (GE PET trace, GE Medical Systems, Uppsala, Sweden). After azeotropic drying, 18F-Fluoride reacted with bromoethyltriflate to yield the radiolabeled synthon 18F-Bromofluoroethane, which, after distillation, reacted with dimethylaminoethanol to give the crude product 18F-Fluoroethylcholine. Purification was achieved by cation exchange (solid-phase extraction) and the final product was obtained after elution with physiological saline. Quality control was performed according to the European Pharmacopoeia [14].

Examinations were performed with the patients in a prone position using a dedicated 16-channel breast coil (Rapid Biomedical, Rimpar, Germany). PET and contrast-enhanced MRI (CE-MRI) acquisitions were performed simultaneously. CE-MRI was performed according to EUSOMA recommendations [15]. The protocol included the following sequences: T2-TSE, STIR, DWI and T1-Dixon-TWIST. T1-weighted sequences were acquired before and after intravenous administration of a paramagnetic contrast agent (Dotarem: 0.2 ml/kg), at a flow rate of 3.5 ml/s. All sequences were acquired in the axial plane.

PET acquisition started immediately after the injection of 2.5–3.5 MBq/kg of 18F-FEC. MRI-based attenuation correction was applied using Dixon-VIBE sequences, with in-phase and opposed-phase, as well as fat-saturated and water-saturated images. A three-dimensional (3D) acquisition technique was used that offered an axial field of view (FOV) of approximately 26 cm and a transverse FOV of 59 cm with a sensitivity of 13.2 cps/kBq.

### Image analysis

Lesions were initially detected and evaluated on CE-MRI of the breast by an experienced breast radiologist (> 8 years of experience). Clearly benign findings on MRI, such as simple cysts or non-enhancing lesions, were discarded. Suspicious findings, requiring histological verification, were selected for further analysis and maximum lesion diameter was measured. The targeted lesion was then correlated with the 18F-FEC PET images and quantitative evaluations were performed by a nuclear medicine physician (> 7 years of experience). 18F-FEC uptake was measured with a dedicated software (Hermes 3D Hybrid Viewer, Hermes Medical Solutions, Stockholm, Sweden) by drawing a region of interest (ROI) on the lesion. In case of a suspicious lesions in the breast, the axillary lymph nodes were also sampled. The lymph node with the highest FEC uptake was considered, regardless of the morphological lymph node characteristics on MRI. The slice in which the breast lesion or axillary finding showed the maximum uptake was selected for the evaluation, and the minimum, mean, and maximum standardized uptake value (SUV_min_, SUV_mean_, SUV_max_) was recorded for each lesion (SUV_T_) and lymph node (SUV_LN_). Readers were blinded to the previous imaging examinations, clinical history of the patient, and final histology, but they were not blinded to the simultaneous CE-MRI findings.

### Histopathological analysis

Included breast lesions and suspicious lymph nodes underwent image-guided core needle or vacuum-assisted biopsy.

In patients who did not undergo neoadjuvant chemotherapy and in whom a sentinel lymph node biopsy was performed, these results were considered the standard of reference for the evaluation of the lymph node status.

For this analysis, lymph nodes with macrometastases (at least one metastasis > 2.0 mm) were considered positive, while lymph nodes with micrometastases (> 0.2 mm and/or > 200 cells but < 2.0 mm) or isolated tumor cells were considered negative [16, 17]. Malignant breast lesions underwent immunohistochemical evaluation of estrogen receptor (ER), progesterone receptor (PR), and HER2 receptor status. If HER2 status was ambiguous, fluorescent in situ hybridization was performed. The MIB-1 monoclonal antibody was used to determine the proliferation activity (expression of the Ki-67 antigen as determined by standard MIB-1 antibodies). All features were dichotomized according to international guidelines [16]. Ki-67 was considered high when > 20%. The material was analyzed by dedicated breast pathologists.

### Statistical analysis

Statistical analysis was performed using dedicated software (IBM SPPS Statistics for Windows, v. 20.0.0, Armonk, NY).

The comparison of the continuous 18F-FEC SUV_maxT_ values between different lesion types and characteristics was performed with either the Mann–Whitney U test (two independent samples) or the Kruskal–Wallis test (three or more independent samples). In addition, a Pearson correlation coefficient matrix was used to assess the correlation between SUV_maxT_, SUV_maxLN_, cancer characteristics, and lymph node status in invasive carcinomas. Partial correlation was used to measure the effect of lesion size.

The diagnostic performance of 18F-FEC SUV_max_ was evaluated using the area under the Receiver Operating Characteristics curve (ROC).

Differences were considered significant at *p* < 0.05. No alpha error accumulation correction was used in this exploratory study. Therefore, interpretation of statistically significant results must consider the possibility of false-positive significances.

## Results

**Fig. 1:**
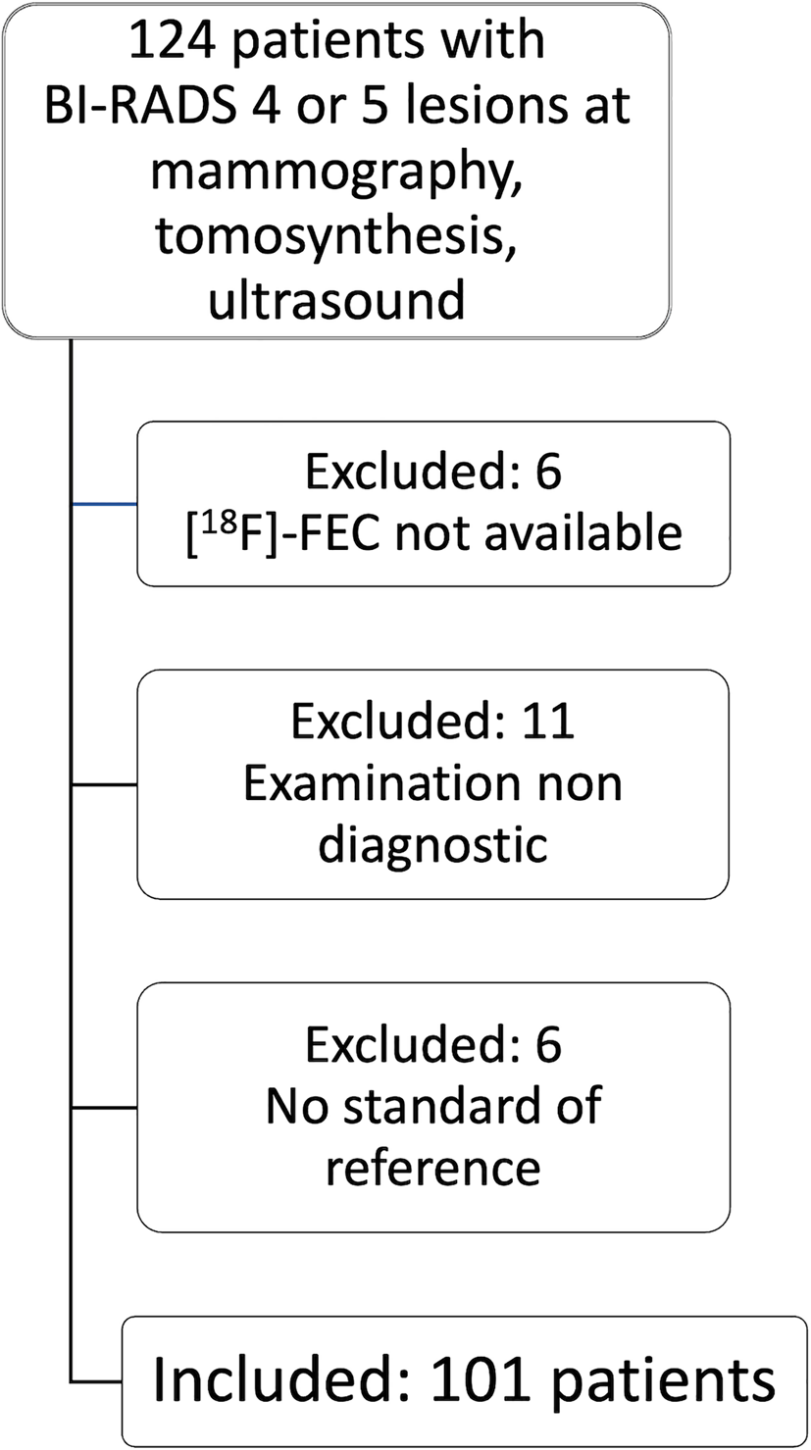
Flowchart showing the cases included and excluded from the study

We included 101 patients (mean age 52.3 years, standard deviation (SD) 12.0, range 30–84) with 117 histologically verified breast lesions (Fig. 1). Thirty lesions were benign, and 87 were malignant (Table 1). Mean lesion size was 2.8 cm for benign lesions (SD 2.4, range 0.6–11.0 cm) and 3.2 cm for malignant lesions (SD 2.5, range 0.6–11.9 cm). A standard of reference for the lymph node status was available for 55 patients. Lymph node metastases were found in 23/55 (41.8%) cases.

**Table 1:** Details on the 117 histologically verified lesions included in the analysis

Histology	Number (%)
Benign	30 (26)
Fibroadenoma	12 (40)
Adenosis, sclerosing adenosis	6 (20)
Papilloma	4 (13)
Fibrosis, fibrocystic changes	3 (10)
Others*	5 (17)
Malignant	87 (74)
Invasive carcinoma NST	40 (46)
Invasive carcinoma NST with DCIS	35 (40)
Invasive lobular carcinoma	5 (6)
DCIS	7 (8)

*NST* non-special type, *DCIS* ductal carcinoma in situ

*PASH, periductal mastitis, hamartoma

All patients tolerated 18F-FEC PET/MRI well, and no adverse events were noted.

### 18F-FEC for the differentiation of benign and malignant breast lesions

**Fig. 2:**
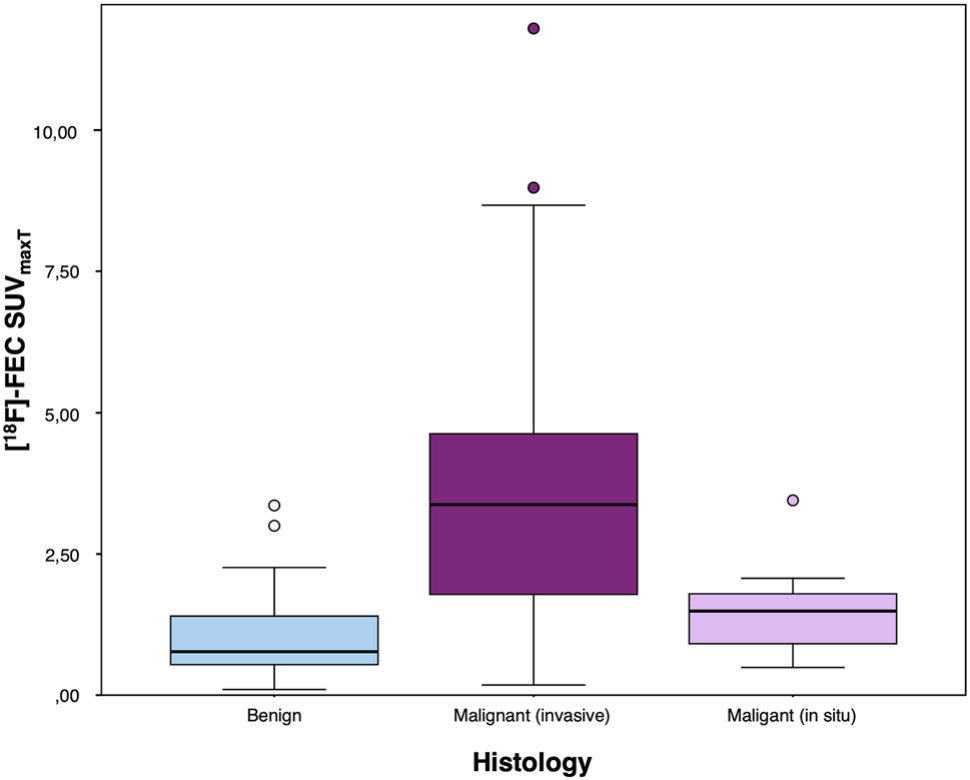
Boxplot showing the distribution of 18F-FEC SUV_maxT_ in benign and malignant lesions. Malignant lesions are divided between invasive and in situ carcinomas

The SUV_maxT_, SUV_minT_, and SUV_meanT_ of 18F-Fluoroethylcholine for benign and malignant lesions are shown in Table 2. The SUV_T_ was significantly higher in malignant than in benign breast lesions (*p* < 0.001, Table 2, Fig. 2).

**Table 2:** Maximum, minimum, and mean standardized tumor uptake values (SUV_maxT_, SUV_minT_, SUV_meanT_) of 18F-fluoroethylcholine for benign and malignant lesions, and for invasive carcinomas (IC) and ductal carcinomas in situ (DCIS) separately

	Benign	Malignant	*p* values	IC	DCIS	*p* values
SUV_maxT_						
Mean (SD)	1.05 (0.79)	3.36 (2.22)	< 0.001	3.51	1.55	< 0.001
Minimum	0.10	0.18		0.18	0.49	
Maximum	3.36	11.8		11.8	3.45	
SUV_minT_						
Mean (SD)	0.69 (0.50)	1.61 (1.25)	< 0.001	1.67	0.86	< 0.001
Minimum	0.05	0.12		0.12	0.18	
Maximum	2.54	7.37		7.37	1.38	
SUV_meanT_						
Mean (SD)	0.87 (0.55)	2.47 (1.65)	< 0.001	2.56	1.21	< 0.001
Minimum	0.08	0.17		0.17	0.25	
Maximum	2.25	9.53		9.53	2.50	

*SD* standard deviation

A significant difference in the SUV_T_ was found between invasive carcinomas and benign lesions (*p* < 0.001) and between invasive carcinomas and DCIS (*p* = 0.009 for SUV_max_, *p* = 0.017 for SUV_mean_, *p* = 0.087 for SUV_min_). No significant difference was found between DCIS and benign lesions nor between ductal and lobular carcinomas (*p* > 0.227 and *p* = 0.092, respectively).

The ROCs of SUV_T_ in distinguishing benign from malignant lesions were: 0.846 (95% confidence interval 0.774–0.918) for SUV_max_; 0.841 (95% CI 0.770–0.912) for SUV_mean_; and 0.764 (95% CI 0.674–0.854) for SUV_min_.

The ROC of SUV_maxT_ in distinguishing invasive carcinomas from DCIS were: 0.797 (95% CI 0.668–0.926) for SUV_max_; 0.772 (95% CI 0.632–0.912) for SUV_mean_; and 0.696 (95% CI 0.539–0.852) for SUV_min_.

### 18F-FEC for molecular subtyping of breast cancers and lymph node status

**Fig. 3:**
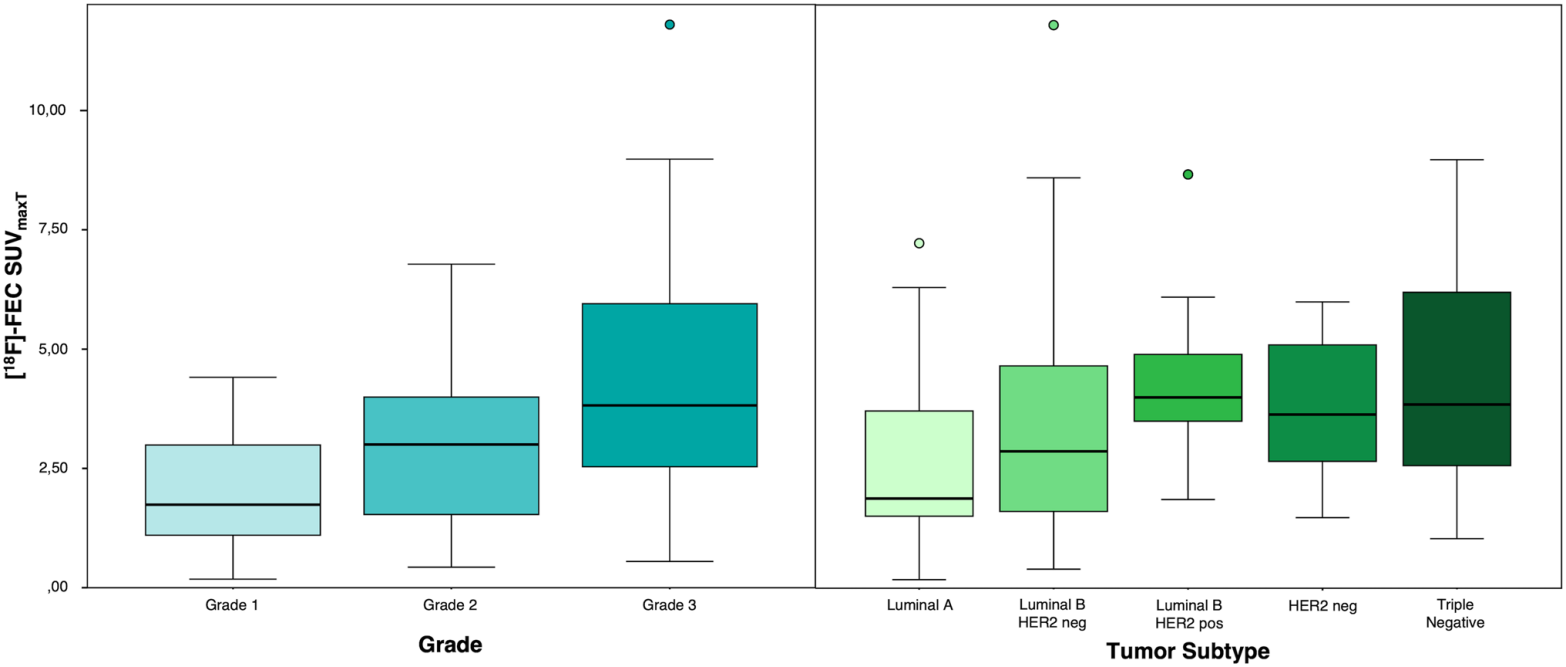
Boxplot showing the distribution of 18F-FEC SUV_maxT_ in malignant invasive carcinomas with different tumor grades and tumor subtypes. A significantly higher 18F-FEC SUV_maxT_ uptake was found in grade 3 cancer, HER2 positive cancers, and in cancers with a high proliferation rate (triple-negative)

18F-FEC SUV_maxT_ increased with tumor grade, positive HER2 status, and high proliferation rate (Tables 3, 4, Fig. 3). More aggressive cancer subtypes (luminal B-HER2 positive, HER2-positive, triple-negative) showed a higher uptake compared to less aggressive tumor types (Table 4). We did not find a significant difference between SUV_maxT_ in ER and PR receptor-positive compared to receptor-negative tumors (Table 3).

**Table 3:** 18F-FEC SUV_max_ levels for estrogen receptor, progesterone receptor, HER2, and Ki-67-positive and -negative tumors

	N° (%)	Mean SUV_max_ (SD)	Minimum SUV_max_	Maximum SUV_max_	*p* value
Estrogen receptor					0.266
Negative	18	3.97(0.50)	1.04	8.98	
Positive	62	3.38(0.29)	0.18	11.8	
Progesterone receptor					0.855
Negative	27	3.85(0.56)	0.18	11.8	
Positive	52	3.34(0.25)	0.40	8.67	
HER-2					0.041*
Negative	60	3.31(0.30)	0.18	11.8	
Positive	20	4.12(0.38)	1.48	8.67	
Ki-67					0.011*
Negative	29	2.67(0.32)	0.18	7.23	
Positive	51	3.99(0.33)	0.40	11.8	

*SD* standard deviation

**Table 4:** Characteristics of the invasive carcinomas included in the analysis

	N° (%)	Mean SUV_max_ (SD)	Median SUV_max_	Minimum SUV_max_	Maximum SUV_max_	*p* value
Grade						0.013
1	9	2.12 (0.49)		0.18	4.41	
2	35	3.03 (0.28)		0.43	6.78	
3	36	4.34 (0.42)		0.18	11.8	
Subtype						0.102
Lum A	23	2.62 (0.37)	1.88	0.18	7.23	
Lum B HER2-neg	29	3.48 (0.47)	2.87	0.40	11.80	
Lum B HER2-pos	13	4.43 (0.46)	4.00	1.86	8.67	
HER2-pos	5	3.78 (0.81)	3.64	1.48	6.00	
TN	10	4.35 (0.79)	3.85	1.04	8.98	

*Lum* Luminal, *neg* negative, *pos* positive, *TN* triple-negative

**Fig. 4:**
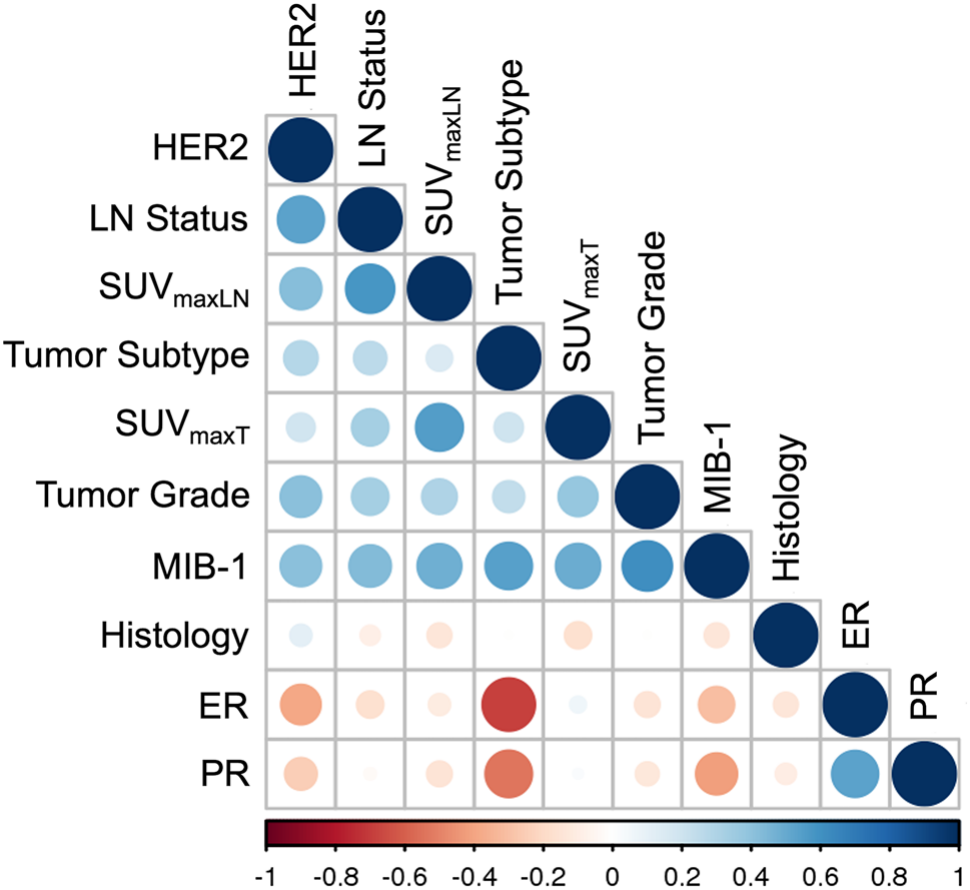
Correlation matrix showing the nonparametric Spearman correlation values (see color-coded lookup bar) between 18F-FEC SUV_maxT_ 18F-FEC SUV_maxLN_ and tumor histology, tumor grade, subtype, lymph node status, estrogen receptor (ER), progesterone receptor (PR), HER2, and Ki-67

A significant correlation between SUV_maxT_ and grade (*r* = 0.356, *p* = 0.001), Ki-67 (*r* = 0.434, *p* < 0.001), lymph node status (*r* = 0.333, *p* = 0.015), tumor histology (*r* = − 0.279, *p* = 0.012), and tumor subtype (*r* = 0.237, *p* = 0.034) was noted (Fig. 4). Partial correlation was used to determine the relationship of tumor grade, subtype, and Ki-67 while controlling for lesion size. A moderate positive partial correlation between SUV_maxT_ and grade, Ki-67, and tumor subtype was detected (*p* < 0.001, *p* < 0.001, *p* = 0.026, respectively). Zero-order correlations also showed a moderate positive correlation (*p* < 0.001, *p* < 0.001, *p* = 0.050), indicating that size had little influence in controlling for the relationship between SUV_maxT_ and these variables.

**Fig. 5:**
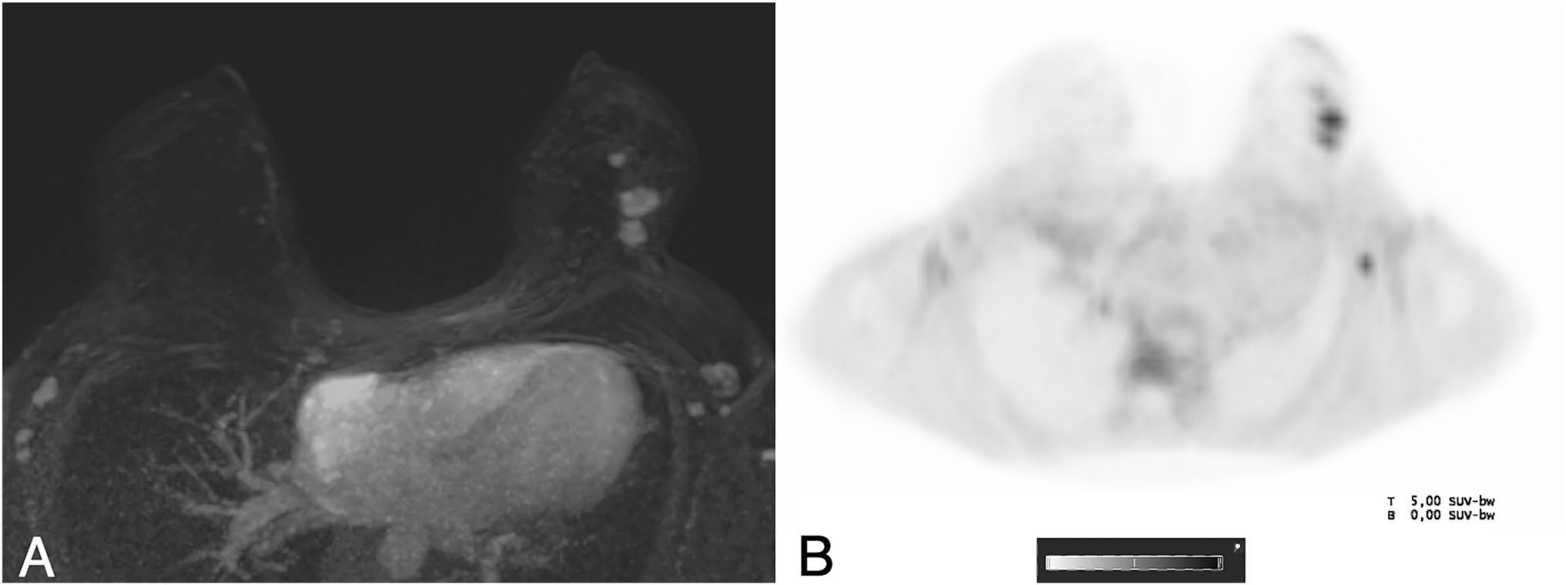
A 42-year-old woman with a suspicious palpable finding in the left breast. Contrast-enhanced MRI revealed multiple suspicious lesions in the upper quadrants of the left breast (**a**, T1 post-contrast subtracted images, Maximum Intensity Projection (MIP), white circle), and asymmetric lymph nodes (white arrow). The lesions (black circle) and the lymph node (black arrow) showed a strong 18F-Fluoroethylcholine uptake (SUV_max_ tumor 3.52, SUV_max_ lymph node 2.69, PET MIP image in **b**). Histology revealed an invasive carcinoma non-special type grade 3, luminal B, HER2-positive, with a high proliferation rate and a metastatic lymph node

**Fig. 6:**
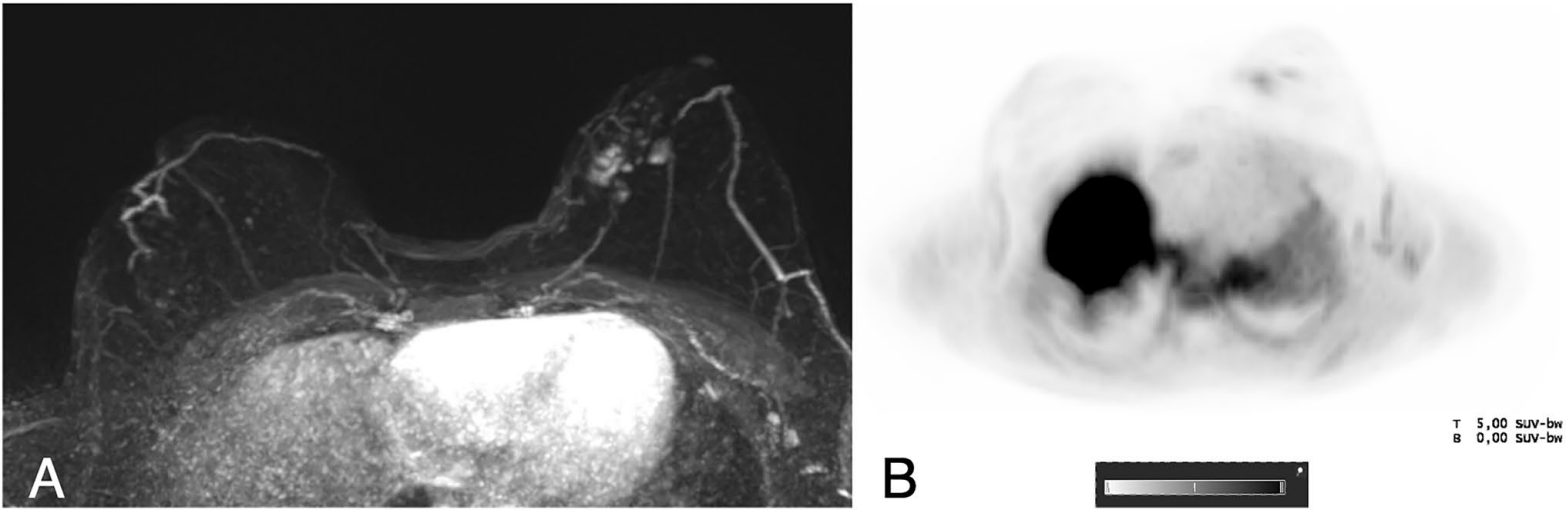
A 51-year-old woman with a suspicious palpable finding in the left breast. Contrast-enhanced MRI revealed a multifocal tumor in the medial quadrants of the left breast (**a**, T1 post-contrast subtracted images, Maximum Intensity Projection (MIP), white circle). Lymph nodes were slightly larger in the left axilla, but with no suspicious morphology (white arrow). The lesion showed a weak 18F-Fluoroethylcholine uptake (SUV_max_ tumor 1.74, PET MIP images in **b**, black circle), as well as a weak uptake by the lymph nodes. Surgery revealed a multifocal invasive carcinoma non-special type grade 2, luminal A with a low proliferation rate and no lymph node metastasis

SUV_maxLN_ showed a significant positive correlation between lymph node status (*r* = 0.576, *p* < 0.001), grade (*r* = 0.296, *p* = 0.032), Ki-67 (*r* = 0.481, *p* < 0.001), and HER2 receptor status (*r* = 0.424, *p* = 0.002) (Fig. 4). Examples are given in Figs. 5 and 6.

The ROC of SUV_maxT_ in distinguishing Ki-67-positive from Ki-67-negative carcinomas was 0.672 (95% CI 0.550–0.794).

The ROC in distinguishing malignant from non-metastatic lymph nodes was 0.761 (95% CI 0.632–0.889) using SUV_maxT_; 0.793 (0.656–0.931) using SUV_maxLN_; 0.790 (0.649–0.931) with SUV_meanLN_; and 0.782 (0.644–0.919) with SUV_minLN_.

The ROC analysis revealed a rule-out criterion if 18F-FEC SUV was measured in the primary tumor: an SUV_maxT_ < 1.5 was observed in only one of 23 (4.3%) positive lymph nodes. The 18F-FEC uptake in the axilla provided a rule-in criterion: only one of 32 (3.1%) negative lymph nodes showed an SUV_maxLN_ of > 2. This criterion achieved a sensitivity of 61% (14/23 positive lymph nodes presented an SUV_maxLN_ of > 2).

## Discussion

Research gaps in breast cancer diagnosis and treatment include a lack of validated imaging biomarkers that can provide a minimally invasive diagnosis and reliable information about breast cancer aggressiveness and metastatic potential [2]. Our study showed that simultaneous 18F-FEC PET/MRI is safe and could provide additional information in the evaluation of benign and malignant breast lesions, particularly in the assessment of breast cancer aggressiveness and prediction of lymph node status.

In this single-center diagnostic trial, we demonstrated that 18F-FEC is well tolerated, with no adverse events, and can, therefore, be directly translated into clinical practice in women with breast lesions. Thus, in addition to the well-accepted indications of the 18F-FEC tracer for prostate cancer, brain, thoracic imaging, liver, and bladder imaging, 18F-FEC can be expanded to the breast as well [18].

Fluorodeoxyglucose (18F-FDG) PET/CT is currently recommended for whole-body staging in ambiguous cases and patients with aggressive breast cancers [19]. In addition, 18F-FDG PET/MRI has been increasingly investigated for its potential role in the characterization and staging of malignant breast lesions [20, 21]. The use of simultaneous 18F-FDG PET/MRI provides detailed metabolic and anatomic data at the same time and can thus significantly improve local and whole-body staging of breast cancer [22, 23].

Nevertheless, several limitations of 18F-FDG must be taken into account, particularly with regard to its use as a quantitative imaging biomarker for breast cancer: some tumor subtypes (such as invasive lobular carcinoma) show a low uptake [24]; the examination can be non-diagnostic in patients with diabetes; and a number of benign lesions show substantial 18F-FDG uptake [25, 26]. In addition, quantitative measurements from 18F-FDG PET are influenced by breast density and, possibly, age [27, 28]. Thus, an alternative tracer, such as 18F-FEC, is desirable [29].

Our analysis showed that the uptake of 18F-FEC differs significantly between benign and malignant lesions, and this difference can be used for diagnostic purposes, with an ROC of 84.6%. Our results, though, also indicate that the accuracy of 18F-FEC is lower than the accuracy of contrast-enhanced MRI alone. The clinical role of PET tracers is most relevant in the staging and phenotyping of cancer aggressiveness [29]. Our results indicate that 18-FEC uptake correlates with lesion characteristics and suggest that it could provide clinically relevant additional information on cancer aggressiveness and lymph node status. A direct comparison with 18F-FDG is limited, as the vast majority of the 18F-FDG PET/MRI studies that focused on the differential diagnosis of breast lesions used qualitative or semiquantitative measurements [30], rather than quantitative measurements, due to the limited accuracy of the latter in 18F-FDG [31].

Securing accurate and reliable information about breast cancer aggressiveness would be most desirable for the management of breast cancer patients. A common issue is the underestimation of an invasive component after the diagnosis of DCIS: up to 36% of biopsy-proven DCIS will be upgraded to invasive cancer when the whole surgical specimen is analyzed [32, 33]. We found that 18F-FEC SUV_max_ values were significantly lower in DCIS compared to invasive cancers and report an exploratory ROC of 79.7 for 18F-FEC SUV_max_ measurements in the identification of invasive cancer, which could potentially reduce DCIS underestimation at biopsy.

Breast cancer staging also relies on the analysis of image-guided biopsy specimens to determine tumor biology [19]. We found an association between tumor grade and 18F-FEC uptake, which is in-line with the results of a pilot 18F-FEC PET study that focused on estrogen-receptor-positive cancers only [11]. We did not find a significantly lower uptake in invasive lobular carcinomas, which has, in contrast, been shown for 18F-FDG [24].

We found a significantly higher 18F-FEC uptake in cancers with an increased proliferation index (Ki-67) and, overall, in more aggressive tumor subtypes. This is similar to what has already been shown for 18F-FDG [34–36]. We did not find an association between 18F-FEC and hormonal receptor status, which has been identified for 18F-FDG [34, 35].

Our study also highlighted the role of 18F-FEC in the assessment of the lymph nodes. The 18F-FEC uptake levels were higher, both in the lesion and in the lymph node, in the presence of lymph node metastasis. The evaluation of lymph node status is essential for therapy optimization but remains one of the major limitations of imaging. Our results indicate that primary cancer lesions with low 18F-FEC uptake have a very low risk of lymph node metastasis, while a significant 18F-FEC uptake in the axilla (SUV_maxLN_ of > 2) is almost exclusively the case in positive lymph nodes. These findings could directly change patient management. While the results of a single study with 18F-FDG PET/MRI [37] were promising, there are conflicting data regarding the usefulness of 18F-FDG for lymph node staging [38, 39], and the topic is still under investigation [23]. Nevertheless, the association between choline metabolism and lymph node status has already been demonstrated by MR spectroscopy [40], suggesting that choline levels correlate with lymph node status and could significantly improve current lymph node staging. 18F-FEC has the advantage of a higher sensitivity compared to MR spectroscopy and it is more reliable and simpler to evaluate in clinical practice.

Our study has some limitations: due to the focus on suspicious BI-RADS 4 and 5 lesions, an expectedly small number of benign lesions were present in our patient population. The prospective character of the study and the preselection of suspicious lesions, as reflected by the high cancer prevalence, reflect real clinical practice, and should, thus, be considered a strength of the study. The reported diagnostic performance metrics are applicable to similar settings and would be rather underestimated compared to settings with fewer suspicious findings. In addition, due to the inclusion criteria, the included lesions were rather large. Further analysis will be necessary to confirm the results in the clinical setting. The limited number of breast cancer subtypes resulted in a low statistical sensitivity to detect potential differences in their 18F-FEC uptake, but the absence of significance should not be interpreted as the absence of differences between subtypes. In this study, CE-MRI images were used only for lesion detection. To evaluate the added value of 18F-FEC together with CE-MRI was beyond the scope of this paper. Lymph nodes in patients with no suspicious breast lesions were not included in the study due to the lack of a standard of reference. This might have resulted in a decrease in the number of false-positive results that could be expected with 18F-FEC.

In conclusion, simultaneous 18F-FEC PET/MRI of the breast allows evaluation of malignant and benign breast lesions with a sufficient accuracy. High 18F-FEC SUV uptake was associated with histopathological features that point toward more aggressive phenotypes and metastatic lymph nodes. Our data indicate the potential of 18F-FEC PET/MRI to be used as a substitute for 18F-FDG in the staging of breast cancer. 18F-FEC PET/MRI has the potential to noninvasively aid in the identification of more aggressive cases, which would profit from more aggressive therapies. Further research focused on whole-body staging with PET/MRI or PET/CT as well as studies directly comparing 18F-FEC and 18F-FDG will be needed to confirm these results and allow the introduction of 18F-FEC as a tracer for breast cancer staging in clinical practice.
